# Evaluation of a real-time optoelectronic method in the diagnostics of CIN over four years of observations

**DOI:** 10.1371/journal.pone.0247702

**Published:** 2021-02-26

**Authors:** Barbara Suchońska, Wanda Gajzlerska-Majewska, Mirosław Wielgoś

**Affiliations:** 1 1stDepartment of Obstetrics and Gynecology, Medical University of Warsaw, Warsaw, Poland; 2 Department of Drug Technology and Pharmaceutical Biotechnology, Medical University of Warsaw, Warsaw, Poland; Texas A&M University, UNITED STATES

## Abstract

Cervical cancer is considered to be particularly amenable to prevention and highly treatable in its early stages. The real-time optoelectronic method of cervix examination seemed to be very promising in the detection of cervical squamous intraepithelial lesions and demonstrated relatively good efficacy. Although this method was introduced into clinics almost 10 years ago, it has not found its place in diagnostic schemes. At the moment, cytological smears and HPV detection with genotyping are still essential. TruScreen seems to be a slightly forgotten test. The aim of the study was to evaluate the efficacy and accuracy of TruScreen in detecting cervical pathology: CIN and cervical cancer confirmed with a histopathological diagnosis in comparison with other methods–cytology and colposcopy over four years of observations. The study was conducted on 130 women with abnormal Pap smear results. We can conclude that a real-time optoelectronic method like TruScreen can be useful as an effective initial cervical cancer screening in developing countries, possibly in combination with other methods. The combination of cytology and TruScreen examination may help clinicians to take decision about the next diagnostics steps (e.g. colposcopy) and contribute to better primary screening for cervical cancer.

## Introduction

Cervical cancer, due to its relatively slow progression of preinvasive lesions, is considered to be particularly amenable to prevention. It usually takes about 10 years for a cervical intraepithelial neoplasia (CIN) lesion to develop into an invasive carcinoma. This type of cancer is highly treatable in its early stages and with early detection and treatment the precancerous lesions, the five-year survival rate for women with CIN is nearly 100% [1]. The ideal screening strategy for the effective detection of cervical intraepithelial neoplasia and early forms of cancer with microinvasion is still needed due to the high incidence of invasive cervical cancer and the poor outcome of advanced disease treatment. Identification of precancerous lesions leads to an effective cure with the use of current methods of treatment. Sometimes, the result of different tests are not compatible. In rare cases, it takes too long to confirm cervical pathology via cervical histopathology. In these cases, only the second or even third investigation performed by a pathologist is correct and perfectly consistent with the actual state.

A perfect screening test is characterised by its simplicity, cost effectiveness, the non-invasiveness of the procedure, accessibility, reproducibility with low false negative rate. A real-time optoelectronic (TruScreen) method seemed to be very promising in the diagnostics of cervical intraepithelial neoplasia (CIN) a few years ago. The advantages of this technology were its immediate and objective results. It was suspected that the method would find its place in clinical procedures, but it has not yet been consulted in any medical association recommendations. At the moment, cytological smears and HPV detection with genotyping are still essential. TruScreen seems to be a slightly forgotten test. The aim of the study was to evaluate the clinical value with the sensitivity and specificity of TruScreen in the diagnostics of CIN confirmed with a histopathological diagnosis over four years of observations. The effectiveness of this method as compared to the traditional methods–cytology and colposcopy–was also assessed.

## Material and methods

This study has been approved by Bioethical Committee of Medical University of Warsaw with the consent number KB//128/2013.

The study was conducted on 130 women aged 18–72 (mean age 36 years) with abnormal Pap smear results. None of the women had had a cervical procedure such as a biopsy or conization within the previous 3 months, radiotherapy, phototherapy, excessive bleeding, the presence of large Nabothian cysts or had been pregnant. The Pap smear results distribution in the group was: 37 cases of ASCUS, 71 cases of LSIL and 22 cases of HSIL/ASC-H. Conventional cytology was drawn at least 4 weeks earlier. They underwent a cervical examination with a TruScreen real-time optoelectronic scanner, and following that, a colposcopy was performed. Guided biopsies were carried out if necessary. Colposcopy-directed biopsy was performed when lesions were detected by colposcopy or the transformation zone was TZ 2 or TZ 3 type (cervical curettage) or when there were other clinical indications for biopsy.

The histopathological result was accepted as a reference standard method. A cervical biopsy was performed on 94 patients with an abnormal colposcopy outcome. The follow-up was carried out for the following four years. As a positive histopathological result, we assumed any histopathological result obtained (cervical biopsy during the first colposcopy, LEEP, biopsy performed during long term-observation).

## Results

**Fig 1:**
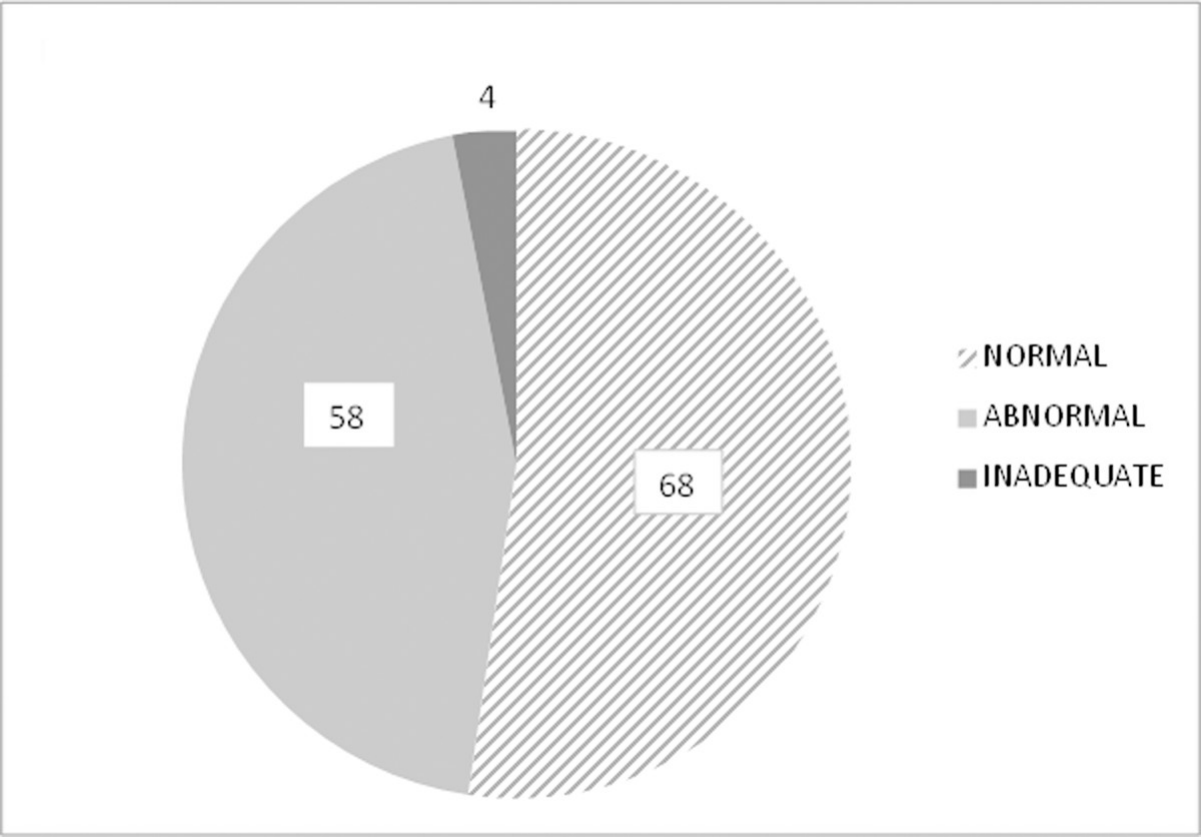
The percentage of normal (52.3%, n = 68) and abnormal (44.6%, n = 58) results of TS in the examined group.

In the study group of 130 women with abnormal Pap smear results, normal results after cervical examination with a TruScreen scanner were found in 68 patients (52.3%), abnormal results were found in 58 patients (44.6%) and 4 results were indeterminable (3.1%) (Fig 1).

CIN lesions were diagnosed in 26 patients with an abnormal TruScreen cervix examination result. Among 14 cases of CIN undiagnosed by TruScreen (negative or inadequate result), there were 8 cases of CIN 1, and 6 cases of CIN 2+. In 4 patients with an abnormal Pap smear, a normal TruScreen result, and a normal histopathology result, CIN 1 developed during the 4-year observation period.

Of the 68 patients with an abnormal Pap smear result, and a normal TruScreen cervix examination result, in 46 cases (68%), CIN lesions were not observed in colposcopy and/or cervix biopsy result.

**Fig 2:**
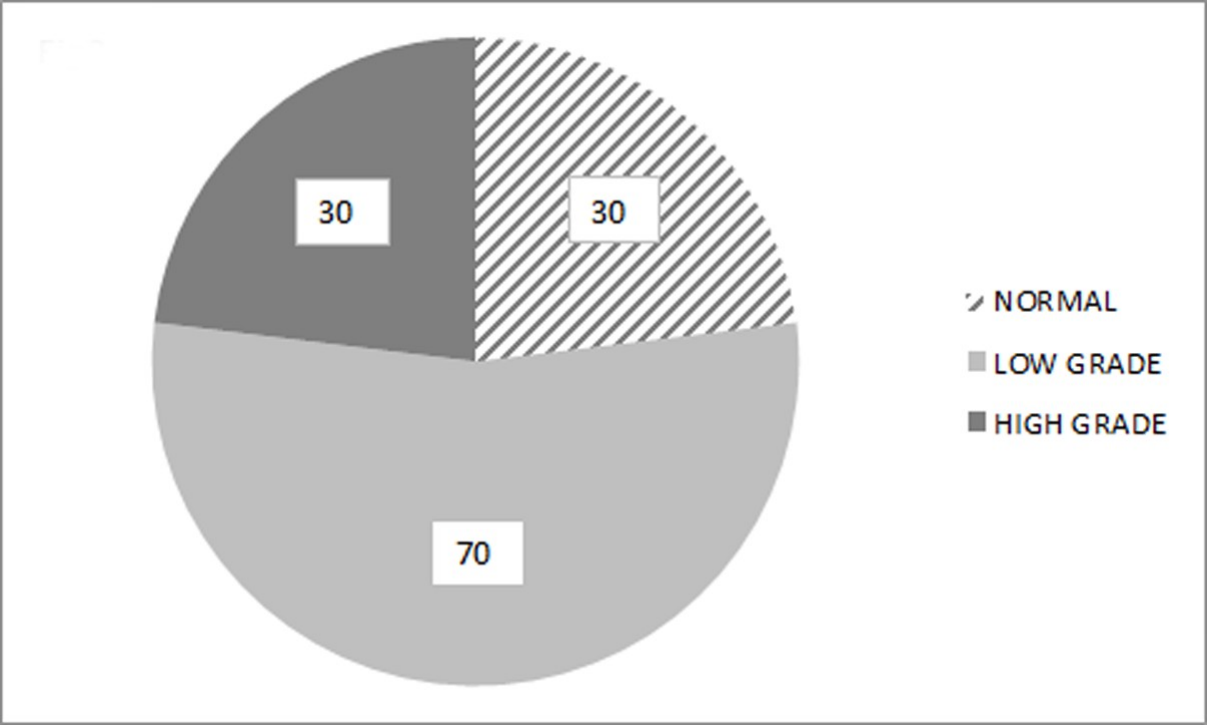
Percentage of normal (23.1%, n = 30), and abnormal (76.9%, n = 100)–low-grade changes (53.8%, n = 70) and high-grade changes (23.1%, n = 30) results of colposcopy examinations.

After a colposcopy examination, normal results were found in 30 patients (23.1%) and abnormal ones in 100 patients (76.9%), with low grade changes in 70 patients (53.8%), and high grade changes in 30 patients (23.1%) (Fig 2).

**Fig 3:**
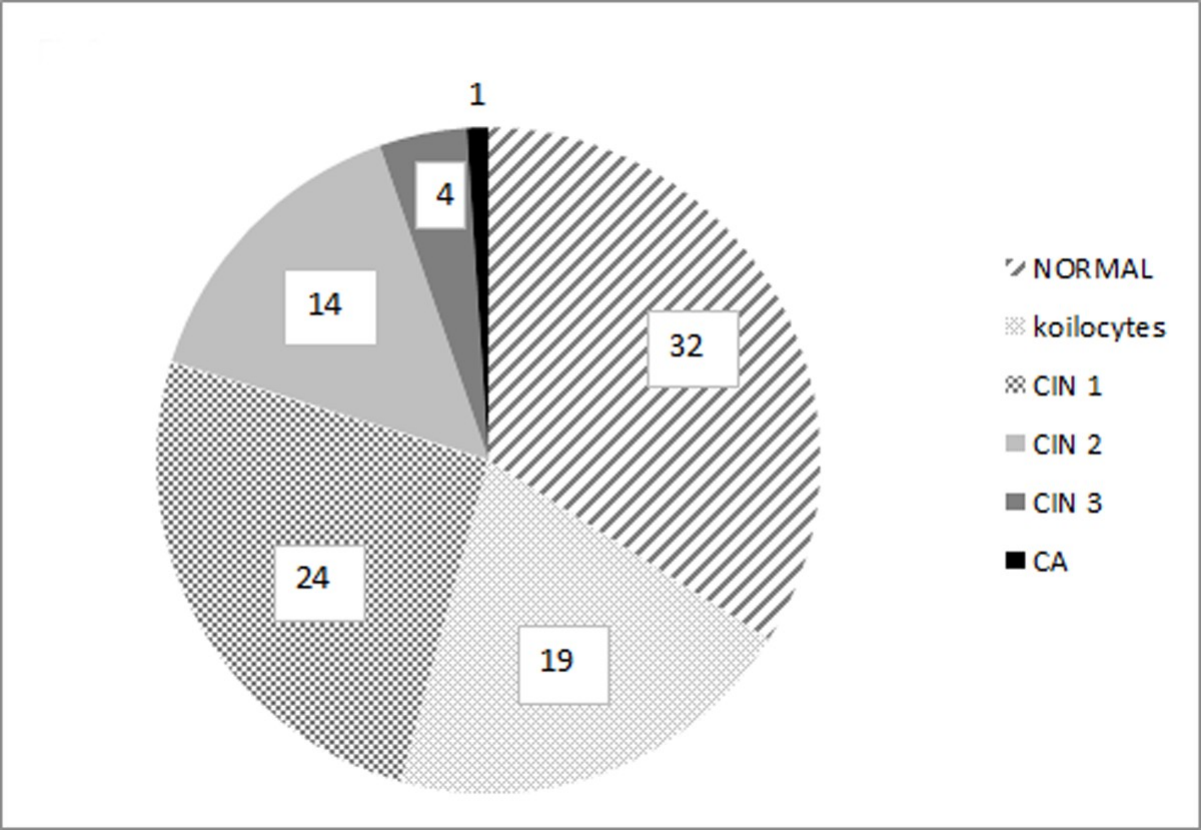
Percentage of normal and abnormal histopathology results in the examined group (n = 94).

Histopathology reported 43 abnormal results: 1 case of cervical cancer, 18 cases of HSIL/CIN2+, and 24 cases of LSIL/CIN1 (Fig 3).

Among the 24 cases of CIN 1, five were diagnosed during our 4-year observation period.

The sensitivity, specificity for abnormal cervical biopsy result and positive predictive value of TruScreen were 65%, 55% and 58% respectively (Table 1).

**Table 1:** Cross table for calculations of sensitivity and specifity of TruScreen for abnormal cervical biopsy results.

		Histopathology result	
		CIN (+)	No CIN (-)
TruScreen	Abnormal (+)	26	19
	Normal (-)	14	32

## Discussion

TruScreen seemed to be a very promising method because it is an automated process and removes the question of subjectivity from the assessment. At present, it is obvious that the optoelectronic method, like cytology and colposcopy, is unable to identify neoplasia in the endocervix. This is an important limitation. Pruski et al. reported that the optoelectronic method doesn’t allow all cases of cervical adenocarcinoma to be detected, whereas a test for the presence of DNA HPV HR allowed all cases of adenocarcinoma and CIN to be recognised [2]. That is the reason that only this method is planned to be used in screening in the future. The optoelectronic method was considered to be effective at detecting precancerous lesions within the squamous epithelium of the cervix.

The result of TruScreen is expressed as normal or abnormal, so the clinician has no detailed and proper information and guidelines to proceed further. A normal results represents a normal squamous epithelium or metaplasia, whereas an abnormal result indicates CIN or cancer.

Coppleson is an author of a classic study from 1994 on using the optoelectronic method in gynaecology [3]. He included women with an abnormal cytological smear or colposcopy result. According to Coppleson, the sensitivity of the optoelectronic method was fairly high—for identifying LSIL/CIN1, it was 88%, while for HSIL/CIN 2+, it was 91%, and 99% for cervical cancer. The specificity of this method was, respectively, 94%, 97% and 86% for LSIL/CIN 1, HSIL/CIN2+ and cancer. These results were much more promising than in our study. Ours are more similar to results conducted by Singer and Coppleson later, in 2003 in Australia and the UK. They were not so enthusiastic. The sensitivity for LSIL lesions detection was estimated at 67% and for HSIL at 70% [4]. The specificity was 81% for CIN and 95% for cancer. In our research we showed that there is a remarkable percentage of false negative results. Those undiagnosed CIN lesions including high grade lesions show limitations of TruScreen method in the diagnostics as a single tool.

According to a meta-analysis published in 2018, based on a Chinese population, the diagnostic accuracy of the TruScreen device is moderately good. The pooled sensitivity and pooled specificity of the TruScreen is 76% and 69% respectively. Because of the moderate accuracy of the device, the authors suggest that the use of this device should be combined with other cervical cancer screening methods to increase the specificity, sensitivity and the clinical value of the TruScreen [5].

In the Polish context, it was proven that the specificity for TruScreen was 82%. The sensitivity was 63% for LSIL /CIN1 and 85% for HSIL /CIN2+ and planoepithelial carcinoma. Pruski et al. presumed that the optoelectronic method was an effective tool for the detection of cervical intraepithelial lesions. According to them, the specificity of a colposcopy for a normal result came to 54%. The sensitivity of colposcopy for LSIL changes was 85% and for HSIL, 97% [6]. In another study of these authors, published in 2011, the advantage of TruScreen over traditional cytology was confirmed. The specificity of the optoelectronic method for LSIL was estimated at 65,7%, and for HSIL, 90.38%. The sensitivity for LSIL lesions was 65.79%, and for HSIL, 90.38%. The above-mentioned data refer to planoepithelial carcinoma. However, the optoelectronic method allowed only 1 in 4 cases of adenocarcinoma to be identified [2].

Besides TruScreen, the sensitivity of the Pap smear changes ranges from 30% to 87% [7, 8]. The sensitivity and specificity of the liquid-based cytological test (LBC) are 73%-94%, and 58%-76%, respectively. It has been reported that 92.9% of cases with a squamous intraepithelial lesion (HSIL) and 100.0% of squamous cell cancer (SCC) were diagnosed by LBC, compared to 77.8% and 90.9% by traditional smear [9–11].

So there are places in the world where TruScreen is used in conjunction with conventional cytological Pap tests to improve accuracy. In Itzkowitc’s analysis, 19.3% of random patients had an abnormal TruScreen result. On colposcopy, 10.3% of the whole group had an abnormal result, while 8.9% had a normal one. A few women with CIN were identified only via TruScreen and a few only via a pap smear. There were women with CIN missed by both methods. In Australia, patients are apt to accept the combined TruScreen and Pap testing approach [12].

In the results of Sung Jong, the false negative rate of Pap smears for CIN was 27.3%, the sensitivity of TruScreen for LSIL/CIN 1 and HSIL/CIN2+ were 75.8% and 77.3%, respectively. A remarkable improvement in the accuracy of the combined test (the combination of TruScreen and cytology) for LSIL/CIN1 (sensitivity 96.8%) and HSIL/CIN 2+ (sensitivity 92.4%) was noted. The combination modalities of TruScreen and cytology were more sensitive for CIN than for cervical cytology alone [13].

According to the results obtained by Singer, Zanardi and Itzkowic [4, 12, 14] and researchers in China [15], the cervical cancer screening combination of TruScreen and cervical smear is of high sensitivity, specificity and consistency with pathological result [16]. When both are normal, the gynaecologist can provide the patient with a high degree of assurance that no significant cervical abnormality is present. But this means the optoelectronic method alone is possibly not good enough in clinical practice.

The experience of Zanardi suggests that TruScreen holds the potential to both detect lesions that might be missed by cytology alone and clarify an unsatisfactory or ASCUS cytology result. 67% of ASCUS that were verified by TruScreen as normal were subsequently confirmed with colposcopy [14].

According to Zhang, his study performed on 187 biopsy specimens indicates that the usefulness of TruScreen is similar to that of traditional methods, liquid-based cytology and HPV HR DNA detecting. Due to the time and logistical issues associated with LBC and HPV testing, TruScreen offers an improved primary screening solution for cervical cancer [17].

According to Global Cancer Data (GLOBOCAN) Report, more than 85% of the global burden of cervical cancer cases and 88% of cervical cancer deaths occur in low- and middle-income countries. In these areas the burden from cervical cancer remains because of the difficulty in implementing cytology-based screening programmes. Due to difficult access to gynaecologists, GPs or other highly skilled technical manpower and a lack of cytodiagnostic laboratories, there is a great need to design cervical cancer screening programmes using alternative strategies. Hence the idea of using visual-based techniques, that are low-cost, have minimal training requirements, give fast analysis and automated features, but are effective and compatible with the prevailing socioeconomic realities [18, 19].

It is important, however, to bear in mind the limitations of this method. The first limitation and disadvantage is the relatively low specificity of the device and high rate of false positive results—possibly leading to nonessential inspection and psychological burden on patients. The other disadvantage, that we should be aware of, is that the device is not good enough at detecting cervical canal lesions. This aspect is of crucial importance in the case of postmenopausal women whose squamous-columnar junction of the cervix is localised in the cervical canal, and in cases of adenocarcinoma [5].

We can conclude that a real-time optoelectronic method like TruScreen can be useful as an effective initial cervical cancer screening in developing countries, possibly in combination with other methods.

## Conclusions

The real-time optoelectronic method of cervix examination demonstrated relatively good efficacy in the detection of cervical squamous intraepithelial lesions. Although this method was introduced into clinics almost 10 years ago, it has not find its place in diagnostic schemes. The combination of cytology and TruScreen examination may help clinicians to take decision about the next diagnostics steps (e.g. colposcopy) and contribute to better primary screening for cervical cancer. Other methods, such as DNA HPV detection and genotyping in cervical scrapes are more useful with higher sensitivity and specificity, so they are supposed to be applied in cervix cancer screening.

## Supporting information

S1 File (ODS)
